# Monocyte chemoattractant protein-1 is not required for liver regeneration after partial hepatectomy

**DOI:** 10.1186/s12950-016-0136-1

**Published:** 2016-08-22

**Authors:** Stephanie L. Wyler, Shawna L. D’Ingillo, Cheri L. Lamb, Kristen A. Mitchell

**Affiliations:** 1 Department of Biological Sciences, Boise State University, 1910 University Drive, Boise, ID 83725-1515 USA; 2 Biomolecular Sciences Ph.D. Program, Boise State University, Boise, ID 83725 USA

**Keywords:** MCP-1, CCR2, Macrophages, Kupffer cells, Partial hepatectomy, Liver regeneration

## Abstract

**Background:**

Liver regeneration following 70 % partial hepatectomy (PH) requires the coordinated expression of soluble mediators produced by macrophages. Monocyte chemoattractant protein-1 (MCP-1) is a potent stimulus of monocyte recruitment and macrophage activation. The goal of this study was to determine how MCP-1 contributes to liver regeneration.

**Methods:**

PH was performed on anesthetized C57Bl/6 (wild type) and MCP-1 knockout mice, and macrophage-produced cytokines and hepatocyte proliferation were measured.

**Results:**

In wild type mice, hepatic MCP-1 protein levels increased 4–6 h after PH, and elevated plasma MCP-1 levels were detected 12 h after PH. Hepatocyte proliferation was comparable in MCP-1 knockout and wild type mice, as was the expression of macrophage-derived cytokines, TNFα and IL-6, and levels of phosphorylated STAT3. The number of CCR2^+^ cells in the liver was similar in MCP-1 knockout and wild type mice, which suggests that other chemokines may recruit CCR2^+^ cells in the absence of MCP-1. Studies with CCR2 knockout mice revealed that hepatocyte proliferation was suppressed ~40 % compared to wild type mice 36 h after PH, but proliferation and liver-body-weight ratios were similar at 48 h.

**Conclusion:**

These findings suggest that MCP-1 is not required for PH-induced liver regeneration, yet the role of CCR2 warrants further study.

## Background

Liver regeneration is a complex physiological process that is influenced by soluble mediators, including cytokines, growth factors and hormones [1]. One of the best-studied models of liver regeneration is based on 70 % partial hepatectomy (PH), in which two-thirds of the liver is surgically resected. Whereas hepatocytes in healthy liver are normally quiescent, removal of a substantial portion of the liver induces compensatory hyperplasia as remaining hepatocytes enter and progress through the cell cycle to restore original organ mass [2]. Hepatocyte proliferation is accompanied by the activation of nonparenchymal cells in the liver, which produce cytokines and growth factors that facilitate hepatocyte cell cycle progression [1].

Liver macrophages (Kupffer cells) are particularly important for the early priming phase of liver regeneration. Following PH, Kupffer cells become activated and produce tumor necrosis factor (TNF)-α, which functions through an autocrine pathway to activate nuclear factor kappa B (NF-kB) and stimulate interleukin (IL)-6 production. Kupffer cell-derived IL-6 acts on hepatocytes to activate the signal transducer and activator of transcription-3 (STAT3) pathway, which upregulates immediate-early gene expression and promotes responsiveness to growth factors [3, 4]. The importance of Kupffer cell-derived cytokine production is underscored by findings that liver regeneration is impaired in the absence of IL-6 [5], TNF-receptor-1 [6, 7] and NF-kB signaling [8, 9]. Furthermore, several studies report that hepatocyte DNA synthesis is either reduced or delayed when Kupffer cells are selectively depleted by administration of liposome-encapsulated dichloromethylene diphosphonate and point to decreased production of proliferative cytokines as at least one possible explanation [8, 10, 11].

The stimulus that initiates Kupffer cell activation and subsequent cytokine production during liver regeneration has not been identified. It has been proposed that enteric lipopolysaccharide (LPS) or other microbial products in the portal circulation may be responsible for activating cytokine production in these cells [12]. Along these lines, several studies report modulation of IL-6 levels after PH in mice that lack Myd88, an adaptor protein for the Toll-like (TLR)/IL-1 family of receptors that detect microbial components [13–15]. However, the finding that type I interferon is not required for liver regeneration suggests that microbial products are not a required stimulus for macrophage activation and cytokine production [15]. Hence, mechanisms of Kupffer cell activation during liver regeneration remain unclear.

In the present study, we investigated the possibility that the production of Kupffer cell-derived cytokines and subsequent liver regeneration are influenced by monocyte chemoattractant protein-1 (MCP-1 or CCL2), a well-known, potent stimulus of macrophage recruitment and activation [16–18]. MCP-1 is a small, secreted protein in the CC family of chemokines that contain two, adjacent cysteine residues [19]. Regulated at the transcriptional level, hepatic MCP-1 expression is induced by a variety of stimuli, including lipopolysaccharide, TNFα and IL-1 [20], as well as toxicants such as carbon tetrachloride and D-galactosamine [1]. In the liver, MCP-1 is produced by hepatic stellate cells as well as biliary epithelial cells and perisinusoidal cells [20, 21]. MCP-1 expression has been shown to increase during chronic hepatitis, inflammation and fibrosis [20–23]. Furthermore, it has been reported that MCP-1 expression directly correlates with the number of infiltrating monocytes and macrophages in the liver [21], and inactivation of MCP-1 attenuates liver injury by inhibiting macrophage recruitment [24, 25].

Given the crucial role of macrophage-derived cytokines during liver regeneration, we hypothesized that MCP-1 is important for this early, priming phase of PH-induced regeneration. In the present study, we used MCP-1 knockout mice, as well as C-C chemokine receptor type 2 (CCR2) knockout mice, to determine the significance of MCP-1 to the early priming phase of liver regeneration, based on endpoints of macrophage-produced cytokines and hepatocyte proliferation.

## Methods

### Animals

Female C57Bl/6 (wild type), MCP-1 knockout (strain B6.129S4-Ccl2^tm1Rol^/J), and CCR2 knockout (strain B6.129S4-Ccr2^tm1Ifc^/J) mice were purchased from the Jackson Lab (Bar Harbor, ME) and used at 8–9 weeks of age. MCP-1 and CCR2 knockout mice are fertile and display no gross physical or behavioral abnormalities. Furthermore, numbers of macrophages (peritoneal, alveolar, and Kupffer cells) in these mice are similar to levels found in wild type mice [26]. Mice were housed in a temperature-controlled facility on a 12-h light/dark cycle with unlimited access to food and water. Wild type and MCP-1 knockout mice were anesthetized with inhaled isoflurane, and 70 % PH was performed as previously described [27]. After euthanasia, blood was collected at the axillary plexus and plasma recovered by centrifugation. Remnant liver tissue was collected and processed as described below. Mice that did not receive PH were used as controls, and data from these animals are included on graphs at the “0 h post-PH” time point. The experimental protocol was approved by the Animal Studies Subcommittee and the Research and Development Committee of the Boise Veterans Affairs Medical Center (Boise, ID), where the animals were housed. All animals received humane care according to the criteria outlined in the “Guide for the Care and Use of Laboratory Animals” prepared by the National Academy of Sciences and published by the National Institutes of Health.

### Measurement of MCP-1 levels

To measure hepatic MCP-1 mRNA levels, total RNA was isolated from frozen liver tissue using an RNeasy Mini Kit (Qiagen, Valencia, CA) according to the manufacturer’s protocol. cDNA was synthesized using SuperScript II reverse transcriptase (Invitrogen, Carlsbad, CA). Transcript levels were measured as previously described [27] by quantitative real-time PCR (qRT-PCR) at the Molecular Genomics Core at the University of Texas Medical Branch (Galveston, TX). To measure MCP-1 protein levels in the liver, frozen liver tissue was homogenized in PBS containing 0.5 % Triton X-100, 0.05 % sodium azide, and protease inhibitors. MCP-1 levels were measured in liver homogenates by enzyme-linked immunosorbent assay (ELISA; Thermo Scientific Pierce, Rockford, IL) and expressed as pg per mg liver protein, based on a modified Lowry protein assay (Bio-Rad, Hercules, CA). Plasma levels of MCP-1 were measured using a Cytometric Bead Array (BD Biosciences, San Diego, CA) according to the manufacturer’s instructions. Unpooled plasma samples were run in duplicate.

### Measurement of TNFα and IL-6 by ELISA

Levels of TNFα and IL-6 were measured in unpooled plasma samples using ELISA kits (Pierce Biotech, Rockford, IL). The assays were performed according to the manufacturer’s instructions, and all samples were run in duplicate. The lower limit of detection for the assays was 50 pg/ml.

### Measurement of STAT3 Activation by western blot

Frozen liver tissue was mechanically homogenized as previously described [27]. Cellular debris was pelleted at 10,000 × *g*, and protein concentration was determined by using a Lowry protein assay (Bio-Rad, Hercules, CA). Homogenates (25–50 μg protein/lane) were fractionated by sodium dodecyl sulfate polyacrylamide gel electrophoresis (SDS-PAGE) and transferred to polyvinylidene difluoride membranes for western blot analysis with anti-STAT3 and anti-phosphorylated STAT3 antibodies (Cell Signaling Technology, Danvers, MA).

### BrdU labeling and detection

To measure hepatocyte proliferation, mice were injected intraperitoneally with 50 mg/kg 5-bromo-2′-deoxyuridine (BrdU; Sigma-Aldrich, St. Louis, MO) 2 h prior to euthanasia. In a separate experiment, continuous BrdU labeling was achieved by administering BrdU in the drinking water (0.8 mg/ml). Water bottles containing BrdU were protected from light and replenished daily until mice were euthanized 6 days postoperatively. After euthanasia, fresh liver tissue was fixed in UltraLight™ fixative (Bi-Biomics, Nampa, ID), paraffin-embedded, and processed for immunohistochemical staining. Tissue sections (5-um) were incubated with a biotinylated anti-BrdU antibody (Invitrogen, Carlsbad, CA) followed by avidin-conjugated horseradish peroxidase and the substrate 3,3′-diaminobenzidine (DAB). Sections were counterstained with hematoxylin. For each animal, at least five random 400X fields were examined, and a total of 800–1000 nuclei were counted. The number of brown (DAB)-stained nuclei (BrdU^+^) was expressed as a percentage of total number of nuclei.

### Detection of CCR2^+^ cells by flow cytometry

Nonparenchymal cells were isolated from the liver and analyzed by flow cytometry. Briefly, livers were perfused with Hank’s Buffered Salt Solution (HBSS) containing 50 mM ethylenediaminetetraacetic acid (EDTA), transferred to RPMI 1640 with 2.5 % FBS, minced into a slurry, and poured through a nylon cell strainer. Samples were centrifuged at 60 × *g* for 1 min at room temperature (RT), and pellets containing hepatocytes were discarded. Samples were then centrifuged at 500 × *g* for 10 min at RT. Resulting pellets were re-suspended in Percoll in RPMI 1640 without FBS and centrifuged at 850 × *g*, 30 min, at room temperature. Pellets were depleted of red blood cells by hypotonic lysis, and remaining cells were resuspended in PBS containing 1 % fetal bovine serum. Cells were then incubated with F_c_-Receptor Block (BD Biosciences, San Jose, CA) for 10 min before staining with a rabbit monoclonal anti-CCR2 antibody (Novus, Littleton, CO) followed by a FITC-conjugated goat anti-rabbit antibody (BD Biosciences, San Diego, CA). Stained cells were analyzed on an Accuri C6 flow cytometer (Ann Arbor, MI). At least 50,000 events (viable cells) were collected from unpooled samples and analyzed using Accuri CFlow Plus software.

### Statistical analysis

Data were analyzed using Prism (version 6.0, GraphPad Software, San Diego, CA). Data were evaluated by a Student’s *t*-test, one-way analysis of variance (ANOVA) followed by a Dunnett’s post-hoc test, or by two-way ANOVA and Bonferroni post-hoc test, depending on the number of variables under consideration. Data were considered significantly different at *p* ≤ 0.05.

## Results

### MCP-1 levels increase after PH

**Fig. 1:**
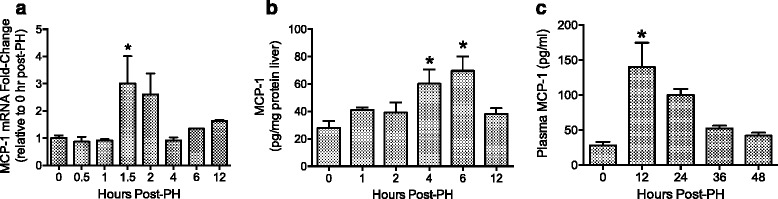
MCP-1 levels increase after PH. **a** MCP-1 mRNA levels in the regenerating liver at the indicated times after PH. MCP-1 mRNA levels (mean +/− SEM) are expressed as fold-induction relative to expression of 18S rRNA in the same samples. Data were measured in three randomly selected samples at each time point, and each sample was run in duplicate. **b** MCP-1 protein levels in the liver after PH. MCP-1 levels (mean +/− SEM) in liver homogenates were normalized to the total amount of protein in each sample (*n* = 5–6). **c** MCP-1 protein levels (mean +/− SEM) in the plasma after PH (*n* = 5–6). * *p* < 0.05 when compared to 0-h group based on one-way ANOVA followed by Dunnett’s test

To examine MCP-1 production after PH, MCP-1 mRNA levels were quantified in the remnant liver, and protein levels were measured in liver homogenates and plasma. Hepatic MCP-1 mRNA levels peaked 90 min after PH (Fig. 1a), followed by increased MCP-1 protein expression in the regenerating liver 4 and 6 h after PH (Fig. 1b). A four-fold increase in circulating MCP-1 was detected in the plasma 12 h after PH (Fig. 1c).

### MCP-1 is not required for the production of TNFα or IL-6 during liver regeneration

**Fig. 2:**
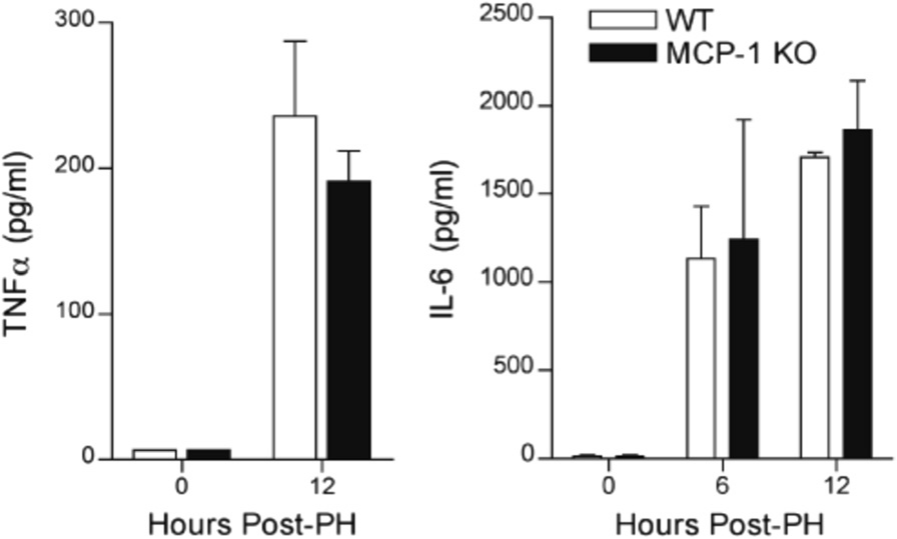
Levels of Kupffer cell-derived cytokines are similar in wild type and MCP-1 knockout mice. Data represent plasma levels (mean +/− SEM) of TNFα and IL-6 in wild type and MCP-1 knockout mice at the indicated times after PH. Cytokines were measured in unpooled plasma samples by ELISA (*n* = 5–6) and run in duplicate. Means at each time point were not statistically significant from each other based on two-way ANOVA

During the priming phase of liver regeneration, the production of TNFα and IL-6 is attributed to activated Kupffer cells [1]. Kupffer cells express the MCP-1 receptor, CCR2, and have been shown to become activated in response to MCP-1 in other model systems [22]. Hence, we hypothesized that MCP-1 may influence the production of Kupffer cell-derived cytokines during liver regeneration. However, measurement of plasma cytokine levels revealed no difference in TNFα or IL-6 production between wild type and MCP-1 knockout mice (Fig. 2). Hepatic mRNA levels of these cytokines were below the limit of detection (data not shown).

### MCP-1 is not required for priming of hepatocytes during liver regeneration

**Fig. 3:**
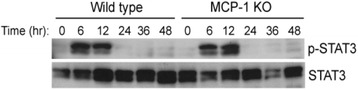
MCP-1 is not required for STAT3 activation during liver regeneration. Western blot analysis of STAT3 and phosphorylated- STAT3 proteins in the regenerating liver of wild type and MCP-1 knockout mice at the indicated times after PH (20 μg protein/lane)

The production of TNFα and IL-6 by Kupffer cells is implicated in priming hepatocytes for cell cycle progression [28]. Upon binding to its cognate receptor on hepatocytes, IL-6 activates STAT3 signaling pathways, leading to gene expression that facilitates hepatocyte proliferation. Hence, STAT3 activation is a pivotal step in regeneration that is likely to be dependent on macrophage activation. Our results indicate that hepatic levels of phosphorylated STAT3 increased 6 and 12 h after PH in both wild type and MCP-1 knockout mice, suggesting that MCP-1 is not necessary for hepatocyte priming during liver regeneration (Fig. 3).

### MCP-1 is not required for hepatocyte proliferation after PH

**Fig. 4:**
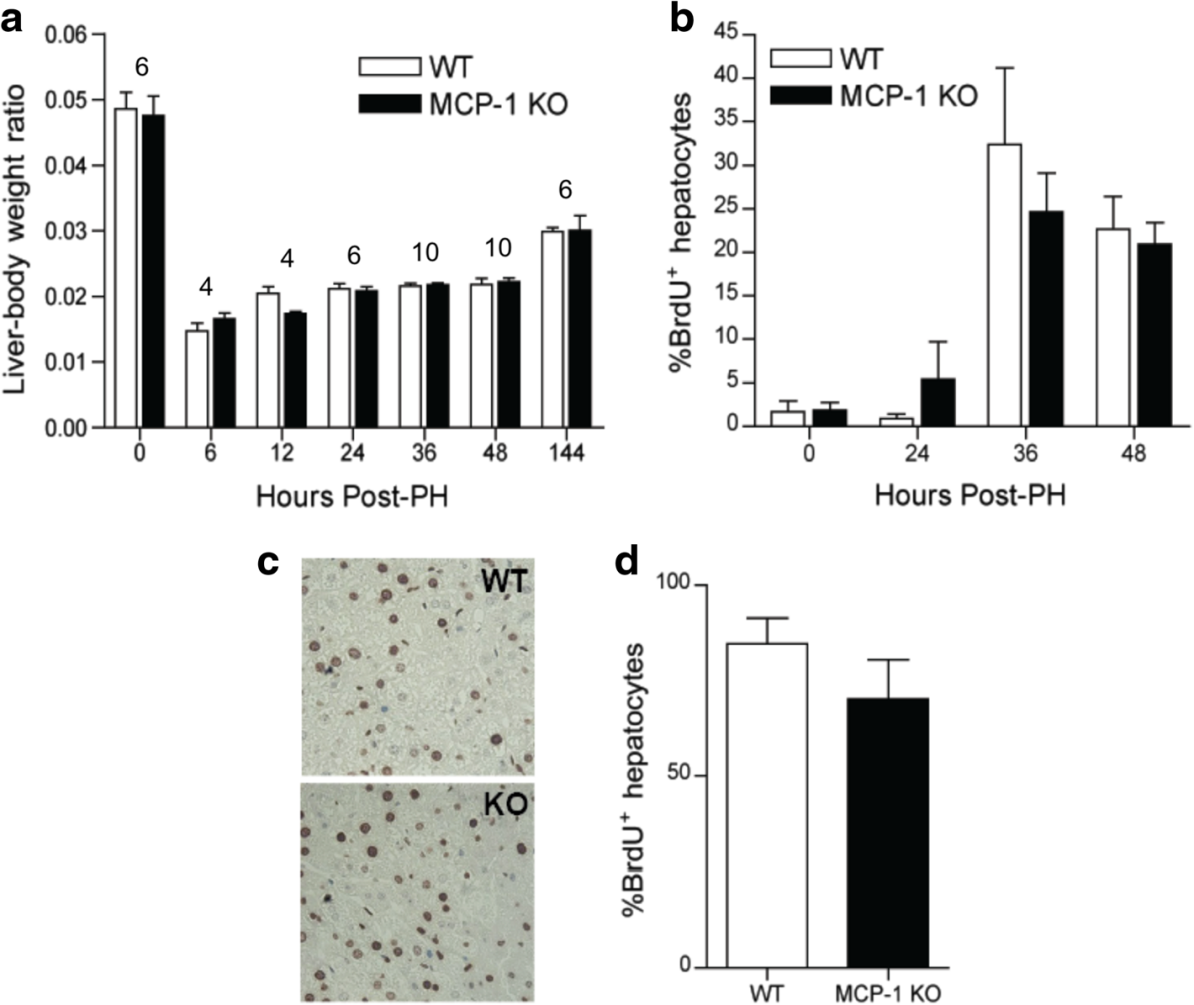
MCP-1 is not required for hepatocyte proliferation after PH. **a** Liver-body weight ratios (mean +/− SEM) of wild type and MCP-1 knockout mice at the indicated times after PH. Sample size varied due to the limited availability of MCP-1 knockout mice. The numbers above the data bars indicate how many MCP-1 knockout mice were used at each time point; an identical number of wild-type mice were used at that same time point. **b** Percentage (mean +/− SEM) of BrdU^+^ hepatocyte nuclei from wild type or MCP-1 knockout mice at the indicated times after PH. Mice were pulsed with BrdU 2 h prior to euthanasia at the indicated time points (*n* = 6 at 0 and 24 h; *n* = 10 at 36 and 48 h). **c** Representative BrdU incorporation in regenerating liver tissue from wild type and MCP-1 knockout mice 6 days after PH; BrdU was added to the drinking water continuously for 6 days. **d** Cumulative BrdU incorporation in the regenerating liver of wild type and MCP-1 knockout mice administered BrdU in their drinking water for 6 days post-PH. Data represent the percentage (mean +/− SEM) of BrdU^+^ hepatocyte nuclei from 4 wild-type mice and 4 MCP-1 knockout mice. Means at each time point were not statistically significant from each other based on two-way ANOVA (Fig. 4 **a**, **b**) or Student’s *t*-test (Fig. 4 **d**)

Finally, we investigated the ramifications of MCP-1 production on the regenerative capacity of the liver. No differences in liver-body weight ratios were detected between wild type and MCP-1 knockout mice (Fig. 4a). Proliferation was measured using BrdU, which is a thymidine analog that is incorporated into newly synthesized DNA. Pulse labeling with BrdU revealed that robust hepatocyte proliferation occurred 36 and 48 h after PH in wild type mice and was not suppressed in MCP-1 knockout mice (Fig. 4b). Likewise, continuous BrdU administration revealed no overt differences in BrdU incorporation in the regenerating liver of wild type and MCP-1 knockout mice 6 days post-PH (Fig. 4c & d).

### MCP-1 is not required for recruitment of CCR2^+^ cells to the regenerating liver

**Fig. 5:**
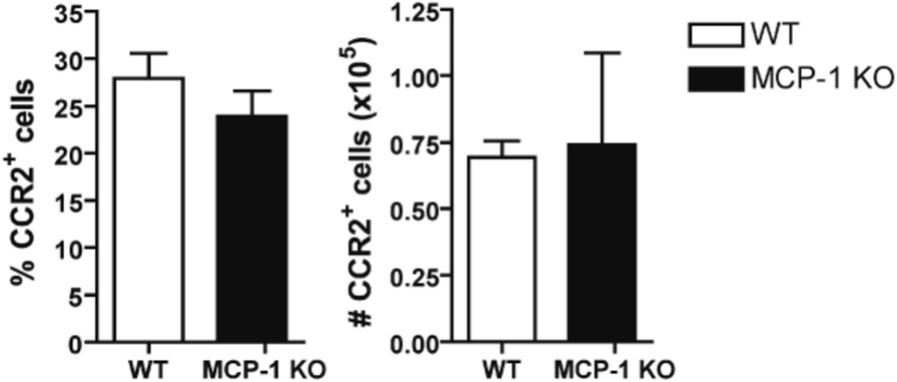
MCP-1 is not required for recruitment of CCR2^+^ cells to the regenerating liver. Flow cytometry was used to measure CCR2 expression in non-parenchymal cells isolated from the regenerating liver of wild type and MCP-1 knockout mice 12 h after PH. Data represent the mean percentage and number (±SEM) of CCR2^+^ cells (*n* = 4). Means were not statistically significant based on Student’s *t*-test

In addition to activating resident macrophages, MCP-1 may also recruit cells, such as bone-marrow-derived monocytes, to the damaged liver [29, 30]. To investigate the possibility that cell recruitment was diminished in the absence of MCP-1, the prevalence of CCR2^+^ cells in the regenerating liver was measured using flow cytometry. The percent and total number of CCR2^+^ non-parenchymal cells in the liver of wild type and MCP-1 knockout mice was comparable 12 h after PH (Fig. 5).

### Hepatocyte proliferation is transiently suppressed in CCR2 knockout mice

**Fig. 6:**
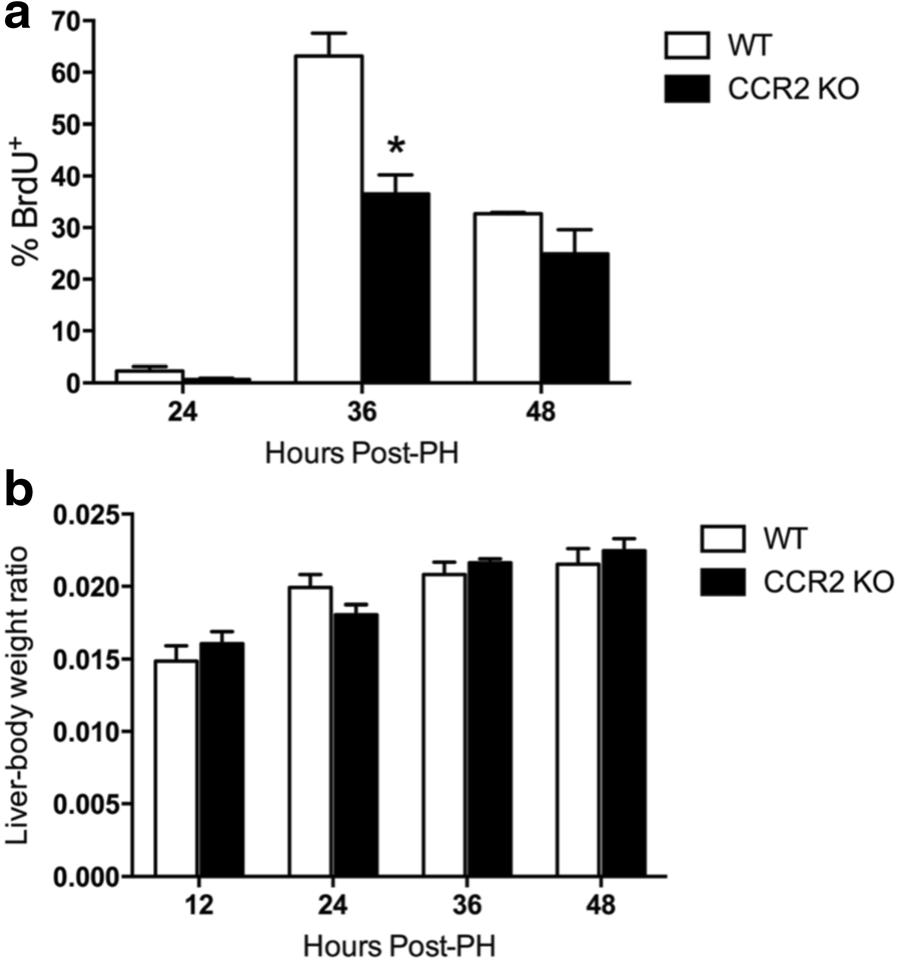
Hepatocyte proliferation is transiently suppressed in CCR2-deficient mice. **a** Percentage (mean +/− SEM) of BrdU^+^ hepatocyte nuclei from wild type or CCR2 knockout mice at the indicated times after PH. Mice were pulsed with BrdU 2 h prior to euthanasia at the indicated time points (*n* = 4). **b** Liver-body weight ratios (mean +/− SEM) of wild type and CCR2 knockout mice at the indicated times after PH. * *p* <0.05 when compared to wild type mice at same time point based on two-way ANOVA followed by Bonferroni’s post-hoc test

While MCP-1 is the most potent ligand of CCR2, there is redundancy in chemokine signaling such that MCP-3 and MCP-5 also bind this receptor, which could explain why liver regeneration was not impaired in the absence of MCP-1. To investigate the collective contribution of all CCR2 chemokines to regeneration, we compared PH-induced hepatocyte proliferation between CCR2 knockout and wild type mice. As shown in Fig. 6, levels of BrdU incorporation were reduced by about 40 % in CCR2 knockout mice 36 h after PH, although no statistically significant suppression was observed at 48 h. Reduced hepatocyte proliferation at 36 h did not coincide with diminished liver-body weight ratios, which remained similar in CCR2 knockout and wild type mice at every time point tested.

## Discussion

Macrophage activation and the production of cytokines are a crucial component of the early phase of liver regeneration following PH [1]. Given the well-characterized role of MCP-1 in activating macrophages and promoting macrophage infiltration, we hypothesized that MCP-1 would be important for this early phase of PH-induced liver regeneration. However, data presented herein refute this hypothesis and demonstrate that, despite increased hepatic levels of MCP-1 after PH, this chemokine is not required for the production of macrophage-derived inflammatory cytokines or for hepatocyte proliferation in the regenerating liver.

Increased MCP-1 expression has been observed in numerous models of liver injury and disease, such as administration of concanavalin A, carbon tetrachloride, and acetaminophen [25, 31, 32]. Our results indicate that hepatic MCP-1 transcript levels increased just 90 min after PH, which corroborates another report [33]. Elevated plasma levels of MCP-1 were detected at 12 h and were only slightly above basal levels. It is possible that plasma levels peaked prior to this time, as it was reported elsewhere that serum MCP-1 levels were robustly elevated at 3 and 6 h after PH in C57Bl/6 mice [34]. However, another study reported only scant plasma levels of MCP-1 in DBA1 mice 4 h post-PH [35].

Although Kupffer cells are a known source of MCP-1, it is likely that multiple cellular sources of MCP-1 exist in the regenerating liver [36]. For instance, in a rat model of PH-induced liver regeneration, elevated MCP-1 mRNA levels were detected in hepatocytes, biliary epithelial cells, sinusoidal endothelial cells, Kupffer cells, pit cells, and hepatic oval cells [37]. It is also possible that MCP-1 production could be attributed to inflammatory cells that infiltrate the liver after PH, such as bone marrow-derived monocytes/macrophages. In fact, an intriguing report by Crane et. al. indicates that F4/80^+^ bone marrow cells are an abundant source of MCP-1, as well as MCP-3 and MCP-5, during infection with murine cytomegalovirus [38]. Hence, during PH-induced liver regeneration, bone-marrow-derived monocytes could be an important source of MCP-1 in the bone marrow, during transit to the liver, or upon infiltration into hepatic tissue.

MCP-1 expression has been shown to directly correlate with the number of infiltrating monocytes and macrophages in the liver [21], and inactivation of MCP-1 reportedly attenuates liver injury by inhibiting macrophage recruitment [24, 25]. However, we found no difference in expression of the macrophage marker F4/80 in wild type and MCP-1 knockout mice (data not shown). Furthermore, plasma levels of TNFα and IL-6 were also similar, which indicates that macrophage activation was likely unaffected by the absence of MCP-1. Studies with bone marrow chimeric mice attribute the PH-induced elevation in circulating levels of IL-6 and TNFα to cells of bone marrow-derived resident macrophages [28, 39]. IL-6 and TNFα signaling pathways are important to the initiation of hepatocyte proliferation after PH, as regeneration is impaired and survival is reduced in IL-6-deficient mice and TNF receptor 1-deficient mice subjected to PH [5–7]. Because no changes in IL-6 were detected between wild type and MCP-1 knockout mice, it would appear that resident macrophages cells are unaffected by the absence of MCP-1 during PH-induced liver regeneration.

During the priming phase of regeneration, IL-6 activates STAT3, which upregulates immediate-early gene expression in hepatocytes and promotes responsiveness to growth factors [3, 4]. Given that levels of STAT3 phosphorylation were similar in wild type and MCP-1 knockout mice, it stands to reason that hepatocyte priming proceeds sufficiently despite the absence of MCP-1. Furthermore, continuous and pulse BrdU labeling revealed similar levels of hepatocyte proliferation in wild type and MCP-1 knockout mice. The observation that liver-body weight ratios were unchanged between wild type and MCP-1 knockout mice further establishes that MCP-1 is dispensable for liver regeneration.

Results from this study demonstrate comparable numbers of CCR2^+^ cells in the liver of wild type and MCP-1 knockout mice, causing us to speculate that other CCR2 ligands, such as MCP-3 or MCP-5, could compensate for the absence of MCP-1. However, the observation that liver regeneration was not overtly suppressed in CCR2 knockout mice renders this notion somewhat irrelevant. In CCR2 knockout mice, hepatocyte proliferation was suppressed 36 h post-PH. However, this did not coincide with decreased liver-body weight ratios, and BrdU incorporation was comparable between CCR2 knockout and wild type mice at 48 h post-PH. The suppression at 36 h may result from diminished macrophage activity in the absence of CCR2, either through decreased monocyte recruitment to the liver or reduced activation of resident Kupffer cells. It has been shown that CCR2 activation in the bone marrow initiates the egress of CCR2^+^ monocytes into the circulation and to the liver [38]. This same report revealed that CCR2^+^ Ly6C^high^ inflammatory monocytes/macrophages accumulated in the bone marrow during MCMV infection, and that the number of inflammatory monocytes/macrophages was diminished in CCR2-deficient mice. Hence, it is possible that monocyte recruitment and/or macrophage activation is reduced in CCR2 knockout mice, leading to a transient suppression of hepatocyte proliferation.

## Conclusions

Liver regeneration is important for maintaining organ mass and function following acute or chronic liver injury. Despite the potent regenerative capacity of the liver, insufficient regeneration remains a significant impediment to recovery from liver disease. Identifying mechanisms that initiate liver regeneration will lead to the development of new therapeutic strategies to enhance recovery from liver disease. Results presented herein rule out a role for the potent macrophage activator, MCP-1, in driving the priming phase of liver regeneration. Furthermore, the observation that regeneration was largely unaffected by the absence of CCR2 indicates that other chemokines that signal through this receptor are probably not crucial regulators of macrophage activation in the regenerating liver. Initiation of regeneration is presumably tightly regulated not only be proinflammatory cytokines, but by other factors that have yet to be identified.

## Abbreviations

BrdU: 5-bromo-2′-deoxyuridine
CCR2: C-C chemokine receptor type 2
DAB: 3,3′-diaminobenzidine
IL-6: Interleukin-6
MCP-1: Monocyte chemoattractant protein
NF-kB: Nuclear factor kappa B
PH: Partial hepatectomy
STAT3: Signal transducer and activator of transcription-3
TNFα: Tumor necrosis factor-alpha
